# Increased HCMV seroprevalence in patients with hepatocellular carcinoma

**DOI:** 10.1186/1743-422X-8-485

**Published:** 2011-10-27

**Authors:** Quentin Lepiller, Manoj K Tripathy, Vincent Di Martino, Bernadette Kantelip, Georges Herbein

**Affiliations:** 1 Department of Virology, University of Franche-Comte, EA 4266, IFR 133 INSERM, CHU Besancon, F-25030 Besançon, France; 2 Department of Hepatology, University of Franche-Comté EA 4266, CHU Besancon, F-25030 Besançon, France; 3 Department of Pathology, CHU Besancon, F-25030 Besançon, France

## Abstract

**Background:**

Hepatocellular carcinoma (HCC) is the most common primary liver cancer, usually arising after years of chronic liver inflammation that could result from viral infections such as hepatitis B virus (HBV) and hepatitic C virus (HCV) infections. Human cytomegalovirus (HCMV) infects primary human hepatocytes and remains an important cause of morbidity in immunocompromised persons where it may manifest as symptomatic end-organ disease including hepatitis. The goal of the present study was to determine a potential correlation between HCMV infection and the appearance of HCC.

**Methods:**

First, we analyzed the seroprevalence of HCMV in a cohort of 11,318 patients hospitalized between 2003 and 2009 in different departments of a French University Hospital. Second, we studied HCMV seroprevalence in a cohort of 190 subjects who were stratified on the basis of age, gender, HCC, cirrhosis (Cir), and the exposition to hepatotropic viruses (HCV, HBV). We further determined whether HCMV DNA was present specifically in tumour area in liver biopsies from HCC-positive patients by using nested PCR.

**Results:**

We found that the HCMV seroprevalence was high in the Hepatology department. The HCMV seroprevalence was significantly higher in patients infected with HCV and/or HBV than in patients who were not infected by those later viruses (76.2% versus 56.5%, p < 0.001). The HCMV seroprevalence was significantly higher in patients with HCC (74%) and lower in patients without HCC (54% for HCC-/Cir+ patients, 57% for HCC-/Cir- subjects). We observed a positive correlation between serum IL-6 levels and HCMV seroprevalence in cirrhotic patients, but not in HCC patients. Serum IL-6 levels correlated positively with C-reactive protein (CRP) levels. Preliminary histological studies from liver biopsies from HCC-positive patients highlighted that HCMV DNA can be detected in tumour area of some of the patients studied.

**Conclusions:**

Our results indicate that HCMV seroprevalence in patients with HCC is significantly higher than in patients without HCC, is positively correlated with serum IL-6 levels in cirrhotic patients, and is positively associated with the presence of other hepatotropic viruses such as HCV and HBV.

## Background

Hepatocellular carcinoma (HCC) is the most common primary liver cancer, usually arising after years of chronic liver inflammation. Hepatitis B virus (HBV) and hepatitis C virus (HCV) infections can lead to HCC, whereas non-infectious inflammatory states, such as chronic inflammation induced by alcohol consumption and hereditary iron overload can also contribute to HCC. IL-6 levels are elevated in the serum of patients with all of these chronic liver diseases [1-3] and could become even more elevated in those who develop HCC [4-6]. Recently, obesity-promoted HCC development was reported to depend on enhanced production of IL-6 which causes hepatic inflammation and activation of the oncogenic factor STAT3 [7].

Human cytomegalovirus (HCMV) is an opportunistic, species-specific herpesvirus that infects a large part of the population worldwide and causes asymptomatic latent infection in healthy subjects. However, it can cause severe disease in the absence of an effective immune response, especially in patients with AIDS and in immunocompromised solid-organ and bone marrow allograft recipients [8]. Histological and immunohistochemical studies have demonstrated the presence of infected cells in virtually all organs and the virus targets a variety of cell types *in vivo*, including macrophages, endothelial cells, epithelial cells, fibroblasts, stromal cells, neuronal cells smooth muscle cells, and hepatocytes [9,10]. Blood monocytes and tissue macrophages are believed to serve as target cells in infected organs, acting as viral disseminators throughout the host or as sites of HCMV latency [11]. Elevated levels of IL-6 have been reported to accompany HCMV replication in transplanted lungs and bone marrow during episodes of inflammation or rejection [12,13]. IL-6 mRNA expression is upregulated by HCMV infection in the absence of *de novo *expression of viral genes [14-17]. Virion binding activates multiple intracellular signal transduction pathways, including the phosphatidylinositol kinase, MAPK/ERK and protein kinase C pathways, all of which lead to the activation of nuclear factors such as NF-kB and p38 [18,19], which are known inducers of IL-6 gene transcription [20].

In the present study, the seroprevalence of HCMV and the IL-6 production were determined in patients hospitalized who were studied on the basis of HCC, cirrhosis and the presence or not of hepatotropic viruses.

## Methods

### Study population and setting

To determine the seroprevalence of HCMV in the departments of the Besancon University Hospital, serum samples were collected for routine serological HCMV diagnostics performed at the Department of Medical Virology, Besancon University Hospital, Besançon, France. The samples were obtained from 11,318 patients hospitalized between 2003 and 2009. We analyzed the data retrospectively considering every patient only once. In addition, fifty adult patients with HCC and cirrhosis, 41 adult patients with cirrhosis but no HCC, and 99 patients with neither HCC nor cirrhosis were eligible for enrolment at the Besancon University Hospital. These patients included a cohort of 92 patients who were participating in studies that assessed HCMV serological analysis, the measurement of serum IL-6 and C-reactive protein (CRP), and clinical outcome. The seroprevalence of HCV, HBV, and HIV was also measured in each serum.

The study was in accordance with ethical principles as formulated in the World Medical Association Declaration of Helsinki.

### Study design

Patients were followed up with use of HCMV serological analysis, HCV, HBV, and HIV serological analysis, the measurement of serum IL-6 and CRP levels and clinical outcome.

### HCMV, HBV, HCV and HIV serodiagnostic

Serum samples were assessed for anti-HCMV IgG and IgM antibodies with use of an ELISA (Elisa Biotest, Liaison Diasorin). Serum samples were also assessed for anti-HCV IgG, HBs Ag, anti-HBs IgG, anti-HBcore IgG and anti-HIV Ig (Axsym).

### Measurement of serum IL-6 and CRP

IL-6 and CRP were measured in the serum of patients using ELISA kit and immunochemistry (R&D systems and Image Beckman Coulter, respectively).

### Histological data

Liver biopsies from 3 patients diagnosed as hepatocellular carcinoma in the Besancon University Hospital were considered for this study. For each patient, the pathologist selected one biopsy in a "tumour area" and another biopsy in a "safe area". All specimens were obtained in paraffin blocks. Prior to DNA extraction, paraffin-embedded specimens were treated with xylene during 5 minutes under agitation and ethanol during 5 minutes under agitation. DNA was extracted and purified using QIAamp kit (Qiagen, Valencia, CA) according to the manufacturer's instructions. The DNA extracted from the liver biopsy was quantified in spectrophotometer. A total of 200 ng DNA was used for performing first PCR reaction using full length primers of *UL82 *(pp71). After initial heating step at 94 for 5 minutes, the thermal cycling protocol was as follows: 1 min at 94°C, 1 min at 60°C and 1.5 min at 72°C for 35 cycles. 10 μl from the 1st PCR product were used in a nested-PCR performed using internal primers for *UL82 *(pp71) in slightly different PCR Conditions: 45 sec at 94°C, 1 min at 60°C and 45 sec at 72°C for 35 cycles. Nested amplification products were visualized on 1% agarose gel electrophoresis and stained with ethidium bromide. The sequences of the primers used were 5'-TAGATGCGGGGTCGACTGCGT-3' and 5'-TCAGGCATCGTCCTCGCCCGG-3' for the *UL82 *(pp71) full length and 5'-CGAAAGCATTCTGGATCTGC-3' and 5'-TTTCTGCATCACGACTCACC-3' for the *UL82 *(pp71) internal length.

### Statistical analysis

Values are the means and SDs of independent experiments. Statistical analysis was performed by student's T test, chi-square and logistic regression for multivariate analyses of factors associated with CMV seropositivity and HCC, and differences were considered significant at a value of p < 0.05. Microsoft Excel was used to construct the plots.

## Results

### High HCMV seroprevalence in several departments including the Hepatology department

To assess the HCMV seroprevalence among patients hospitalized in the Besancon University Hospital and to compare the HCMV seroprevalence between the different hospital departments, serum samples were collected for routine serological HCMV diagnostics performed at the Department of Medical Virology, Besancon University Hospital. The samples were obtained from 11,318 patients hospitalized between 2003 and 2009. We analyzed the data retrospectively considering every patient at least once; hence the number of samples tested is 12,889 (see Additional File 1, Table S1). The HCMV seroprevalence was 50.73% (95%CI: 49.81-51.65) with a mean age of 45.13 years (95%CI: 44.69-45.57). We observed a positive correlation between the age and the HCMV seroprevalence (r^2 ^= 0.616) with a weak HCMV seroprevalence in pediatric and neonatal populations (20.32% and 38.09%, respectively). Among the departments with the highest HCMV prevalence were the Dermatology Department (74.84%), the Urology/Nephrology Department (69.16%), the Infectious Diseases Department (63.81%) and the Hepatology Department (71.88%) (see Additional File 1, Table S1). Among outclinic patients hospitalized in the Dermatology Department and in the Infectious Diseases Department 77.02% and 56.19% respectively, were infected with HIV. The HCMV seroprevalence among patients hospitalized in these two Departments was significantly higher in HIV-positive patients than in HIV-negative patients with 83.06% versus 47.30% (p < 0.001) in the Dermatology Department and 84.75% versus 36.96% (p < 0.001) in the Infectious Diseases Department (Table 1). Among patients of the Hepatology Department, infection with HCV and/or HBV was observed in 36.77% of the patients. The HCMV seroprevalence was significantly higher in patients infected with HCV and/or HBV than in patients who were not infected by those later viruses (76.2% versus 56.5%, p < 0.001) (Table 2).

**Table 1:** HCMV seroprevalence and HIV infection in the Department of Dermatology and of Infectious Diseases.

Department	HIV	Patients	HCMV seropositivity (%)	Age (years, mean)
**Dermatology**	Positive	248	83.06	42.37
	
	Negative	74	47.30	42.27

**Infectious Diseases**	Positive	118	84.75	39.61
	
	Negative	92	36.96	36.09

**Table 2:** HCMV seroprevalence and HCV and/or HBV infection in the Department of Hepatology.

Department Hepatology	HCV and/or HBV	Patients	HCMV seropositivity (%)	Age (years, mean)
**Division 1**	Positive	30	80.0	49.7
	
	Negative	34	64.7	41.3

**Division 2**	Positive	49	81.6	58.5
	
	Negative	99	58.6	57.4

**Division 3**	Positive	52	78.8	54.6
	
	Negative	133	57.9	55.3

**Total**	Positive	164	***76.2***	55.0
	
	Negative	446	***56.5***	53.3

### Higher HCMV seroprevalence in HCC-positive patients

Since we observed a high HCMV seroprevalence in the Hepatology Department, we then focused our study on 50 adult patients with HCC and cirrhosis (HCC+), 41 adult patients with cirrhosis but no HCC (HCC-/Cir+), and 99 patients with neither HCC nor cirrhosis (HCC-/Cir-) who were eligible for enrolment at the Besancon University Hospital. These patients included a cohort of 92 patients who were participating in studies that assessed HCMV serological analysis, serum IL-6 and CRP measurement, and clinical outcome. We matched patients of each group for age and gender. Male subjects were 87% of the patients and the mean age was 66 years.

**Figure 1:**
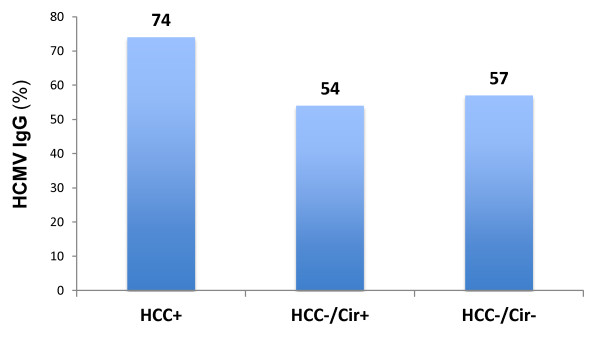
**HCMV seroprevalence in HCC+/Cir+, HCC-/Cir+, HCC-/Cir-subjects**.

HCMV seroprevalence was significantly increased in the HCC+ group versus the HCC-/Cir+ and HCC-/Cir- groups (74% versus 54% and 57%, respectively) (Figure 1). Using unimodal statistical analysis, the HCMV seroprevalence was significantly higher in the HCC+ patients than in the HCC-patients with 74% and 56%, respectively (p = 0.023) (Table 3, Figure 1). The HCMV seroprevalence was not significantly higher in patients with cirrhosis or infected with HCV, HBV or HIV (Table 3). By contrast, HCC was significantly associated with HCV infection (p = 0.046) and always associated with cirrhosis (Table 3).

**Table 3:** Factors associated with HCC and with HCMV seropositivity.

Unimodal analysis							
HCMV associated factors							

	**HCC**	**Cirrhosis**	**HCV**	**HBV**	**HIV**	**Male gender**	**Exposition to hepatotropic viruses**

**p**	***0.023***	0.24	0.23	0.57	0.83	0.09	0.21

HCC associated factors							

	**HCMV IgG+**	**Cirrhosis**	**HCV**	**HBV**	**HIV**	**Male**	**Exposition to hepat. viruses**

**p**	***0.023***	***0***	***0.046***	0.55	0.26	0.21	0.08

**Multimodal analysis**							

HCMV associated factors							

	**Odds Ratio**			**CI95%**	**p**		

**HCC**	3.45			1.34-8.93	***0.010***		

**Cirrhosis**	0.71			0.31-1.62	0.418		

**Age > 65 years**	0.59			0.31-1.14	0.119		

**Exposition to hepatotropic viruses**	1.35			0.62-2.94	0.448		

**Male gender**	0.43			0.15-1.24	0.118		

HCC associated factors							

**IgG HCMV+**	2.76			1.30-5.86	***0.008***		

**Age > 65 years**	1.48			0.73-2.98	0.278		

**Exposition to hepatotropic viruses**	2.07			0.96-4.45	0.064		

**Male gender**	2.80			0.84-9.30	0.093		

Unimodal analysis and Multimodal analysis.

Using multimodal statistical analysis, the HCMV seropositivity was 3.45-fold more frequent in HCC+ patients (OR = 3.45; CI95%: 1.34-8.93; p = 0.010) and was independent on the presence of cirrhosis or on infection with HCV or HBV (Table 3). HCC was positively associated with HCMV seropositivity (OR = 2.76, CI95%: 1.30-5.86, p = 0.008) (Table 3).

### Systemic levels of IL-6, C-reactive protein, HCMV seroprevalence and HCC

**Figure 2:**
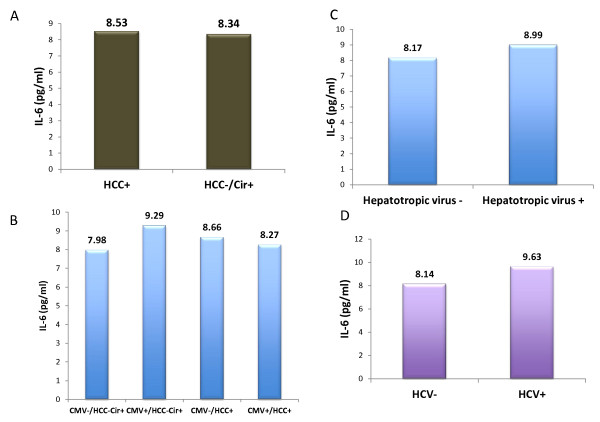
**Serum IL-6 levels in HCC+/Cir+ and HCC-/Cir+ groups**: (A) without taking into account the virological status; (B) infected or not with HCMV; (C) infected or not with hepatotropic viruses (HBV and/or HCV); (D) infected or not with HCV.

**Figure 3:**
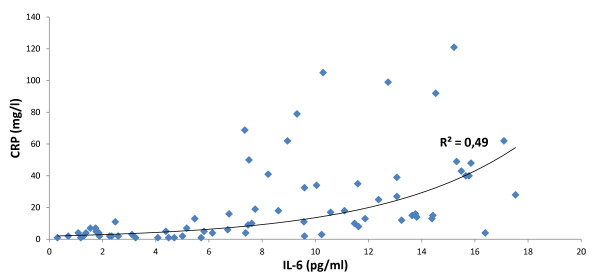
**Correlation between serum IL-6 and CRP**.

HCC development is dependent on enhanced production of the tumor promoting cytokines such as IL-6 which causes hepatic inflammation and activation of the oncogenic transcription factor STAT3 [7,21]. Therefore, we measured serum IL-6 levels. High levels of serum IL-6    were measured in both HCC+ and HCC-/Cir+ groups (means of 8.53 and   8.34 pg/ml, respectively), but no significant difference between the    two groups was observed (p = NS) (Figure 2A). Serum IL-6 levels were significantly increased in the HCC+ and the HCC-/Cir+ groups versus healthy subjects (p < 0.05) (data not shown). Interestingly, the serum IL-6 levels were higher in HCC-/Cir+ patients who were HCMV seropositive (9.29 pg/ml versus 7.98 pg/ml), and the converse was observed in HCC+ patients in whose decreased serum IL-6 levels were measured in HCMV seropositive subjects (Figure 2B). Increased serum IL-6 level was observed in patients infected with hepatotropic viruses (HBV and/or HCV) (8.99 versus 8.17 pg/ml, respectively) (Figure 2C), or with HCV (9.63 versus 8.14 pg/ml, respectively) (Figure 2D). Finally, serum IL-6 correlated positively with that of CRP (r^2 ^= 0.49) (Figure 3).

### Detection of HCMV DNA in the liver biopsies of some HCC-positive patients

**Figure 4:**
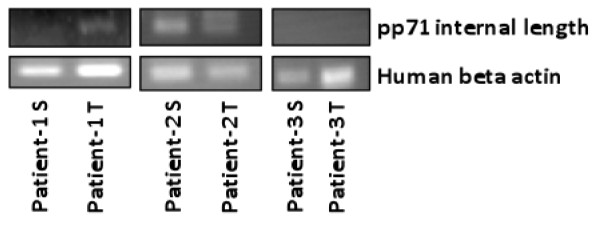
**Detection of HCMV DNA by nested PCR in the liver of HCC-positive patients**. S: safe area, T: tumour area.

Liver biopsies from 3 HCC-positive patients were considered. For each patient, the pathologist selected paraffin-embedded liver biopsy sample both from the tumour area and the safe area. DNA was amplified by nested PCR for HCMV pp71 (*UL82*) gene (Figure 4). Three different results were obtained. HCMV DNA was specifically detected in liver tumour area (T) but not in safe area (S) for one patient (patient 1). In the other patients, HCMV DNA was either not detected (patient 3) or detected in both tumour area and safe area (patient 2).

## Discussion

We report here that HCMV seroprevalence in patients with HCC is significantly higher than that of cirrhotic patients without HCC. We observed a positive correlation between HCMV seroprevalence and serum IL-6 levels in cirrhotic patients, but not in HCC patients. In addition serum IL-6 levels correlated positively with CRP levels. Our results question the role of HCMV in the development of HCC.

HCMV is a highly transmissible and prevalent beta herpesvirus [22,23]. This pathogen is never cleared from the body, persisting in a number of tissues via hypothesized mechanisms including chronic productive infection and/or latent infection with periodic subclinical reactivation [8,22]. Recently, HCMV has been linked to a variety of chronic diseases with an inflammatory component including cardiovascular disease [24], cognitive decline including vascular dementia [25], functional impairment [26] and cancer [27]. Although HCMV has been reported as involved in hepatitis [28], its role in cancer is just starting to emerge. The involvement of HCMV in late inflammatory complications underscores its possible role in inflammatory diseases and cancer. Evidence of this involvement of HCMV in such phenomena is being accumulated (review in: [27,29,30]. Early *in vitro *studies suggested that HCMV was able to transform embryonic fibroblasts in culture and to induce chromosomal damages and mutations but HCMV has never been accepted as oncogenic virus [27]. Later on the concept of "oncomodulation" was proposed to explain the possible contribution of HCMV in tumour progression [31]. The oncomodulation states that HCMV infects the tumour tissue and acts as a cofactor in amplifying mechanisms of carcinogenesis without necessarily initiating tumour. Support for this idea is based on experiments showing that proteins of HCMV (or non-coding RNAs) can influence the genesis and tumour growth acting on the cell cycle, apoptosis, genetic instability, invasiveness, angiogenesis, adhesion and cell migration. These proteins have been the subject of extensive recent reviews [27,32,33]. The increased sensitivity of detection of HCMV in tumour tissues (immunohistochemistry, *in situ *hybridization and PCR techniques) originally proposed by Cobbs *et al *in 2002 served to highlight the presence of HCMV proteins and DNA in tumour cells but not in adjacent cells of several cancers such as glioma [34], colon cancer [35], prostate cancer [36], and some skin cancers [37]. Interestingly, the presence of HCMV was also highlighted in the pre-cancerous lesions such as colorectal polyps [35], and prostatic intraepithelial neoplasia [36].

In order to determine the role of HCMV as a potential oncomodulator of HCC, we measured HCMV seroprevalence in patients with HCC, in cirrhotic patients and HCC-/Cir- subjects. Our results clearly indicate that HCMV seroprevalence is increased in patients with HCC. Using multimodal statistical analysis, the HCMV seropositivity was 3.45-fold more frequent in HCC+ patients and was independent on the presence of cirrhosis or on infection with HCV or HBV. Also, HCC was positively associated with HCMV seropositivity and with infection with HCV or HBV. These results suggest that HCMV could be involved in the appearance of HCC, as it is the case for HCV and HBV. To confirm this hypothesis future studies will determine whether HCMV genome and/or HCMV antigens can be detected in hepatocytes of HCC patients. It is also possible that high HCMV seroprevalence observed in HCC patients and not in cirrhotic patients could result from HCMV infection of vessels of the liver and/or from infected circulating monocytes arriving at sites of liver inflammation [9,38].

We also observed a positive correlation between HCMV seroprevalence and serum IL-6 levels in cirrhotic patients, but not in HCC patients. Increased serum IL-6 levels have been reported in cirrhotic patients and HCC patients infected with HBV and HCV [39-41]. Nevertheless, serum IL-6 levels in HCC patients depend on the virus involved (HBV versus HCV), the genotype of the virus especially for HCV and ultimately from the IL-6 gene polymorphism [42-45]. Elevated levels of IL-6 have been reported to accompany HCMV replication in transplanted lungs and bone marrow during episodes of inflammation or rejection [12,13]. IL-6 mRNA expression is up-regulated by HCMV infection [15-17] and increased IL-6 levels are a critical factor involved in inflammation and carcinogenesis, especially in liver [7]. IL-6 production has been detected in many cell types, however the primary sources of the cytokine are monocytes and macrophages at sites of inflammation during acute inflammation, as well as T cells in chronic inflammation. In homeostatic conditions, IL-6 levels are low, whereas under stress conditions, amounts of IL-6 rise quickly in the serum. Production of IL-6 depends on several transcription factors, primarily NF-kB, C/EBPβ (formerly NF-IL6) and AP-1 [46]. The stimuli that trigger activation of these transcription factors include TNFα and IL-1, bacterial products (LPS) or viral infections such as HIV, HCV and HCMV [47-49]. After IL-6 is secreted from the activated monocyte or macrophage, it can act on other cells locally or systemically. In classical IL-6 signaling, the cytokine engages its receptor IL-6R at the cell surface. IL-6R is a non-signaling receptor normally present only on hepatocytes and certain leukocytes [50]. A homodimer of the signal-transducing receptor gp130 is recruited to the IL-6-IL-6R complex, followed by activation of Janus kinase (JAK) which is recruited to the intracellular portion of gp130. JAK in turns activates the transcription factor STAT3 (signal transducer and activator of transcription) [48,51] by phosphorylation. Phosphorylated STAT3 dimerizes and travels to the nucleus, there initiating a transcriptional program [50]. This program's primary function is to promote growth and differentiation and prevent apoptosis, and includes among others the induction of genes encoding cyclin D1, survivin and Bcl-2 [52]. In addition, STAT3 regulates genes that promote angiogenesis through regulation of vascular endothelial growth factor (VEGF) [53]. The IL-6 signal also activates the mitogen-activated protein kinase (MAPK) pathway, specifically extracellular signal-related kinase (ERK) through JAK activation of SHP2 (a protein-tyrosine phosphatase), which eventually activates the proto-oncogene Ras, a GTPase found mutated in many human cancers [50], especially in HCC [54]. In addition, IL-6 antagonizes TGF-β-induced apoptosis in human hepatoma cell line Hep3B [55], and this could be critical during cirrhosis favoring the transformation of hepatocytes parallel to the development of liver fibrosis. In addition, proinflammatory cytokines are part of the host response to HCMV infection and participate in viral clearance. The finding that HCMV seropositivity correlates with serum IL-6 levels in HCC-/Cir+ patients and not in HCC+ patients suggest that IL-10 which is produced during HCMV infection (vIL-10) does not impair the control of HCMV, but rather may inhibit local production of proinflammatory cytokines such as IL-6 by infiltrating lymphocytes in target organs. Our results are in accord with the observation of Cheeran et al. [56], who reported similar MCMV viral loads in IL-10 deficient mice and in immunocompetent mice, C57BL/6 (which is a model simulated by the IL-10 KO mice repleted with mrIL-10).

HCMV is not an uncommon feature in patients with liver diseases and may be a cause of systemic inflammation. Plasma cytokine interleukin-6 (IL-6) is mainly produced by circulating and peripheral cells and induces the hepatic synthesis of C-reactive protein (CRP), which is the main acute phase reactant. CRP expression *in vivo *is assumed to be restricted mainly to the liver [57] where CRP is produced under the control of various proinflammatory cytokines such as interleukin 1 (IL-1), IL-6 and tumor necrosis factor α (TNFα) [58]. Since IL-6 has been reported to accompany HCMV replication [12,13], IL-6 could favor CRP production in the liver of HCMV-infected patients. Our results are in agreement with this observation, since serum IL-6 correlated positively with that of CRP. Nevertheless, we cannot exclude that CRP production is increased in these patients by bacterial infections that could also favor IL-6 production.

To determine whether HCMV DNA was specifically present in liver tumour area, we further performed a nested PCR for HCMV *UL82 *(pp71) gene. Interestingly, HCMV DNA was specifically detected in the tumour area but not in safe area for one patient among 3 considered patients as previously described for glioma [34], prostate cancers [36], colon cancers [35], and skin cancers [37]. However, HCMV DNA was either detected in both tumour and safe areas or not detected for the other two patients, indicating that the detection of HCMV DNA in tumour area is not an invariant result during hepatocellular carcinoma with our experimental conditions. One limitation of this experiment might be the use of fixed and paraffin embedded biopsies, as such treatment was described to impair DNA [59]. Histological evaluation of more liver biopsies might further be considered to confirm these results.

Some limitations of the current study should be considered. First, the diagnosis of HCMV infection was based on an indirect test and the absence of anti-HCMV-IgG with the detection of HCMV DNA can occur, especially in immunocompromised patients. Second, the measurement of soluble receptor IL-6 might have been helpful as an indirect marker of cell hepatic responsiveness to IL-6 and therefore of CRP expression. Third, the patients were characterized with only one measurement of IL-6 and CRP.

## Conclusions

Our results indicate that HCMV seroprevalence in patients with HCC is significantly higher than in patients without HCC and is positively correlated with serum IL-6 levels in cirrhotic patients. If HCMV infection plays a significant role in the etiology of HCC, the elimination of HCMV infection via the development and administration of treatments or vaccines [60] may reduce HCC mortality rates. Colugnati et al. predicted that a vaccination against HCMV would not need to have high efficacy nor wide-spread coverage to make a substantial impact on HCMV transmission, and elimination of HCMV from the population has the potential to greatly reduce the incidence of disease attributable to HCMV infection [61]. Therefore, elimination of HCMV infection is a potentially feasible and important avenue of study for preventing diseases linked to HCMV infection. Future studies will be needed to further define the role of HCMV in cancers including HCC.

## List of abbreviations

HCC: hepatocellular carcinoma; HBV: hepatitis B virus; HCV: hepatitic C virus; HCMV: human cytomegalovirus; HIV: human immunodeficiency virus; Cir: cirrhosis; CRP: C-reactive protein; IL-6: interleukin-6; JAK: Janus kinase; STAT3: signal transducer and activator of transcription 3; MAPK: mitogen-activated protein kinase; ERK: extracellular signal-related kinase; NF-kB: nuclear factor kappa b; TNF-α: tumor necrosis factor α; TGF-β: transforming growth factor β; LPS: lipopolysaccharide; SD: standard deviation.

## Competing interests

The authors declare that they have no competing interests.

## Authors' contributions

Conceived and design the experiments: QL, VDM, BK, GH. Performed the experiments: QL, MKT. Analysed the data: QL, MKT, GH. Wrote the paper: GH. All the authors have read and approved the final manuscript.

## Supplementary Material

Additional file 1 **Table S1**. HCMV seroprevalence in departments of a French University Hospital. Click here for file
