# Supplementary material for: Increased HCMV seroprevalence in patients with hepatocellular carcinoma
Source: Virol J. 2011 Oct 27;8:485. doi: 10.1186/1743-422X-8-485 (PMC3224784; doi:10.1186/1743-422X-8-485)
Supplement: Additional file 1 — Table S1. HCMV seroprevalence in departments of a French University Hospital. [file 1743-422X-8-485-S1.DOC]

|  | **Department** | **HCMV seropositivity** | **Population** | **HCMV seropositivity (%)** | **Age**  **(years, mean)** |
| --- | --- | --- | --- | --- | --- |
| **1** | DERMATOLOGY Division 1 | 241 | 322 | 74.84 | 42.35 |
| **2** | HEPATOLOGY Division 1 | 46 | 64 | 71.88 | 45.27 |
| **3** | UROLOGY /NEPHROLOGY mixed division | 148 | 214 | 69.16 | 57.87 |
| **4** | HEMODIALYSIS | 39 | 57 | 68.42 | 60.77 |
| **5** | GERONTOLOGY Division 1 | 21 | 31 | 67.74 | 84.74 |
| **6** | NEPHROLOGY Division 1 | 206 | 308 | 66.88 | 56.67 |
| **7** | HEPATOLOGY Division 2 | 98 | 147 | 66.67 | 57.76 |
| **8** | PNEUMOLOGY Division 1 | 39 | 59 | 66.10 | 60.78 |
| **9** | HEPATOLOGY Division 3 | 117 | 180 | 65.00 | 55.2 |
| **10** | INFECTIOUS DISEASES Consulting | 134 | 210 | 63.81 | 38.07 |
| **11** | GYNECOLOGY / OBSTETRICS Consulting | 38 | 60 | 63.33 | 30 |
| **12** | NEPHROLOGY Division 2 | 68 | 108 | 62.96 | 58.98 |
| **13** | GERONTOLOGY Division 2 | 39 | 63 | 61.90 | 85.4 |
| **14** | NEPHROLOGY Intensive care unit | 30 | 50 | 60.00 | 56.76 |
| **15** | ONCOLOGY | 37 | 63 | 58.73 | 52.57 |
| **16** | RHEUMATOLOGY Division 1 | 72 | 124 | 58.06 | 50.67 |
| **17** | NEPHROLOGY Consulting | 115 | 201 | 57.21 | 48.62 |
| **18** | SURGICAL INTENSIVE CARE UNIT | 45 | 79 | 56.96 | 42.25 |
| **19** | DERMATOLOGY Division 2 | 107 | 189 | 56.61 | 53.02 |
| **20** | HEMATOLOGY Division 1 | 244 | 436 | 55.96 | 56.22 |
| **21** | HEPATOLOGY Division 4 | 69 | 126 | 54.76 | 51.98 |
| **22** | RHEUMATOLOGY Consulting | 20 | 37 | 54.05 | 40.57 |
| **23** | OTORHINOLARYNGOLOGY | 35 | 65 | 53.85 | 40.37 |
| **24** | INTERNAL MEDICINE Division 1 | 52 | 97 | 53.61 | 51.27 |
| **25** | HEMATOLOGY Division 2 | 24 | 45 | 53.33 | 63.42 |
| **26** | HEPATOLOGY Division 5 (Intensive Care Unit) | 45 | 85 | 52.94 | 53.28 |
| **27** | OBSTETRICS Division 1 | 167 | 316 | 52.85 | 25.19 |
| **28** | DIGESTIVE SURGERY Division 1 | 19 | 36 | 52.78 | 50.53 |
| **29** | EMERGENCY Division 1 | 31 | 59 | 52.54 | 41.63 |
| **30** | MEDICAL INTENSIVE CARE UNIT | 76 | 145 | 52.41 | 55.63 |
| **31** | INTERNAL MEDICINE Division 2 | 148 | 283 | 52.30 | 55.52 |
| **32** | OPHTALMOLOGY Consulting | 18 | 35 | 51.43 | 51.74 |
| **33** | OBSTETRICS Division 2 | 20 | 39 | 51.28 | 29.02 |
| **34** | INTERNAL MEDICINE Consulting | 112 | 219 | 51.14 | 44.98 |
| **35** | HEMATOLOGY Consulting | 1172 | 2313 | 50.67 | 56.42 |
| **36** | DIGESTIVE SURGERY Division 2 | 16 | 32 | 50.00 | 45.16 |
| **37** | CARDIOLOGY Division 1 | 54 | 108 | 50.00 | 53.26 |
| **38** | CARDIOLOGY Intensive care unit | 76 | 153 | 49.67 | 48.37 |
| **39** | INTERNAL MEDICINE Division 3 | 108 | 220 | 49.09 | 41.69 |
| **40** | GYNECOLOGY | 23 | 47 | 48.94 | 32.32 |
| **41** | CARDIOLOGY Division 2 | 26 | 54 | 48.15 | 52.67 |
| **42** | EMERGENCY Division 2 | 69 | 144 | 47.92 | 47.81 |
| **43** | OPHTALMOLOGY | 131 | 276 | 47.46 | 45.08 |
| **44** | NEUROLOGY | 56 | 118 | 47.46 | 49.29 |
| **45** | HEMATOLOGY Division 3 | 101 | 213 | 47.42 | 57.35 |
| **46** | HEMATOLOGY Intensive care unit | 69 | 147 | 46.94 | 42.91 |
| **47** | PNEUMOLOGY Division 2 | 17 | 37 | 45.95 | 58.24 |
| **48** | PNEUMOLOGY Division 3 | 15 | 33 | 45.45 | 57.88 |
| **49** | INFECTIOUS DISEASES | 176 | 396 | 44.44 | 34.9 |
| **50** | CARDIOLOGY Emergency | 15 | 34 | 44.12 | 43.03 |
| **51** | GASTROENTEROLOGY | 80 | 182 | 43.96 | 44.99 |
| **52** | EMERGENCY Division 3 | 39 | 93 | 41.94 | 45.76 |
| **53** | DERMATOLOGY Division 3 | 31 | 75 | 41.33 | 48.36 |
| **54** | NEONATOLOGY | 43 | 105 | 40.95 | 0 |
| **55** | UROLOGY Consulting | 12 | 30 | 40.00 | 38.13 |
| **56** | OCCUPATIONAL MEDICINE | 29 | 75 | 38.67 | 26.56 |
| **57** | PEDIATRICS Consulting | 24 | 63 | 38.10 | 8.54 |
| **58** | PEDIATRIC HEMATOLOGY/ONCOLOGY Division 1 | 22 | 67 | 32.84 | 8.31 |
| **59** | NEONATOLOGY / PEDIATRICS Intensive care unit | 17 | 53 | 32.08 | 2.87 |
| **60** | PEDIATRIC EMERGENCY Division 1 | 21 | 78 | 26.92 | 5.22 |
| **61** | PEDIATRIC HEMATOLOGY/ONCOLOGY Consulting | 10 | 38 | 26.32 | 14.95 |
| **62** | PEDIATRICS Division 1 | 21 | 80 | 26.25 | 6.24 |
| **63** | PEDIATRIC EMERGENCY Division 2 | 19 | 74 | 25.68 | 5.51 |
| **64** | PREMATURE | 11 | 49 | 22.45 | 1.94 |
| **65** | PEDIATRIC HEMATOLOGY/ONCOLOGY Division 2 | 31 | 140 | 22.14 | 7.47 |
| **66** | PEDIATRICS Division 2 | 11 | 52 | 21.15 | 2.35 |
| **67** | PEDIATRIC EMERGENCY Division 3 | 34 | 164 | 20.73 | 4.13 |
| **68** | PEDIATRICS Division 3 | 25 | 123 | 20.33 | 6.82 |
